# How organizational support promotes teacher professional recognition: a perspective on teachers’ autonomous learning and teaching abilities

**DOI:** 10.3389/fpsyg.2026.1705446

**Published:** 2026-06-16

**Authors:** Jianwen Guo, Han Liu, Jun Wang, Lihan Shen, Sihan Qin, Wenlong Tian

**Affiliations:** 1 College of Elementary Education, Capital Normal University, Beijing, China; 2 Jiaozhou No. 3 Middle School, Qingdao, Shandong, China; 3 College of Education, Minzu University of China (MUC), Beijing, China

**Keywords:** organizational support, professional recognition, autonomous learning ability, teaching ability, teacher

## Abstract

**Introduction:**

Teacher professional recognition is a key factor in promoting teacher professional development and improving educational quality. In reality, many teachers feel undervalued and lack the recognition and attention they deserve, and organizational support often remains superficial. Existing research has mostly focused on either organizational support or teacher competence in isolation, lacking a systematic exploration of their synergistic effects. This theoretical blind spot has limited the academic understanding of the mechanisms of teacher professional recognition and has led to school support measures that are not targeted enough to meet the real needs of teachers, potentially further weakening their sense of recognition and hindering the continuous improvement of educational quality.

**Methods:**

To fill this gap, this study conducted a questionnaire survey of 3,983 primary and secondary school teachers from different regions in China. Data analysis was performed using Mplus 8.0, SPSS 27.0, and Smart PLS 4.0 software.

**Results:**

Organizational support not only directly influences teacher professional recognition but also indirectly affects it through the parallel mediating roles of autonomous learning ability and teaching ability.

**Discussion:**

This mechanism integrates both organizational and individual levels, indicating that organizational support can directly impact professional recognition and revealing two specific mediating paths. The first is the autonomous learning ability path, where organizational support promotes teachers’ active learning and reflection. The second is the teaching ability path, where organizational support enhances teachers’ teaching design and enthusiasm. These multiple paths collectively drive the deepening of teacher professional recognition. Theoretically, this study clarifies the mechanism by which organizational support influences teacher professional recognition, expands the integrated framework of organizational support theory and self-determination theory, and enriches the existing theoretical knowledge system. Practically, the study suggests that schools should establish a comprehensive support system covering policies, leadership, and colleagues, implement differentiated support strategies, and innovate evaluation and incentive mechanisms to genuinely enhance the importance placed on teachers. Future research could adopt longitudinal or experimental designs, expand sample diversity and representativeness, and integrate qualitative evidence to enhance the validity and robustness of research results.

## 1 Introduction

Teacher professionalization is a common goal pursued by educators worldwide. It is widely believed that teacher professional training programs on a global scale will promote the professionalization process of teachers (Amirova, 2020). From an individual teacher’s perspective, professional development is essentially about teacher learning (Lieberman and Pointer Mace, 2008), which can be understood as a dynamic process where teachers achieve self-improvement and professional recognition through continuous learning. Existing research indicates that the formation of a teacher’s professional identity is influenced by both personal experiences (such as educational background and teaching practice) and organizational environment (such as school culture and leadership; Flores and Day, 2006). Thus, both individual and social factors significantly impact a teacher’s attainment of professional recognition.

On the one hand, according to the Organizational Support Theory (OST; Eisenberger et al., 1986), the positive influence of organizational support on teachers’ professional recognition is evident. The professional growth of teachers cannot be achieved without the assistance and support provided by the school organization. If schools can offer teachers opportunities for collaboration and professional assistance, encourage them to frequently participate in collaborative activities, jointly prepare lessons, and receive systematic professional training, it will foster a sense of common purpose and emotional investment among teachers, strengthen their connection with the school organization (Yin and Zheng, 2018), and promote their professional development. However, in reality, the majority of teachers worldwide feel undervalued (Akiba et al., 2023), lacking recognition and importance, and organizational support often remains superficial. Although many schools support teacher development, the resources, systems, and emotional support they actually provide are often fragmented and superficial, such as the lack of follow-up after training programs and the lack of transparency in study leave allocation (Salifu et al., 2025).

On the other hand, teachers are both knowledge disseminators and lifelong learners. Their autonomous learning ability and teaching ability are key components of their professional identity and growth. Firstly, autonomous learning ability is an indispensable key ability for teachers’ development. In the context of rapid development of big data and artificial intelligence, if teachers stop learning, they are highly likely to be replaced by artificial intelligence and left behind by the times. For instance, during the pandemic, teachers had to quickly learn online teaching skills to be competent in their work. Secondly, teaching ability is a fundamental ability for teachers. In the education context dominated by exam-oriented education in China (Meng et al., 2021), teachers need to possess excellent teaching skills to help students master knowledge, improve their scores, and thereby facilitate their admission to top schools. This is a crucial step in evaluating whether a Chinese teacher is qualified or successful. Therefore, teachers’ autonomous learning ability and teaching ability are crucial factors for their continuous professional recognition.

From a theoretical perspective, there are obvious gaps in existing research. Most of the existing literature focuses on the impact of organizational support on teachers’ job satisfaction, organizational commitment, or job burnout (Aban et al., 2019; Oubibi et al., 2022), with relatively few studies directly addressing its effect on the core identity variable of “professional recognition.” More crucially, although scholars have separately explored the significance of autonomous learning ability or teaching ability for teacher development (Antoniou and Kyriakides, 2013; Abou Said and Abdallah, 2024), few studies have incorporated organizational support and teacher competencies into the same analytical framework to examine their mechanisms in the formation of professional recognition. The lack of consideration for the dual roles of individual capabilities and organizational support has led to existing theories being unable to fully explain the formation process of teachers’ professional recognition: Is it organizational support that directly shapes recognition, or does it indirectly play a role by empowering teachers’ intrinsic capabilities? Do the paths of different capability dimensions differ? These theoretical blind spots limit our systematic understanding of the mechanisms of teachers’ professional recognition and also make practical interventions lack precise theoretical guidance, making it difficult to effectively enhance teachers’ confidence, innovative vitality, and teaching quality.

Therefore, this study aims to reveal the internal mechanism of organizational support affecting teachers’ professional recognition through a large-sample questionnaire survey, thereby providing a systematic theoretical basis and practical strategies for enhancing teachers’ professional recognition.

## 2 Literature review and hypotheses

### 2.1 Theoretical framework

The Organizational Support Theory (OST) was first proposed by Eisenberger et al. (1986), representing a successful application and deepening of the Social Exchange Theory in the field of organizational behavior research. OST mainly emphasizes employees’ perception of how the organization values their contributions and cares for their well-being, and how this perception influences their attitudes and behaviors (such as commitment, performance, and retention). According to this theory, when teachers feel supported by the organization in terms of salary, professional development platforms, and promotion opportunities, they will develop a sense of obligation to reciprocate based on the principle of reciprocity, thereby demonstrating higher loyalty, dedication, and retention intentions (Rhoades and Eisenberger, 2002; Bogler and Nir, 2012), which helps teachers gain professional recognition. At the same time, the Self-Determination Theory (SDT) further explains how organizational support can be transformed into individuals’ intrinsic motivation and behavioral engagement. When external conditions meet individuals’ needs for autonomy, competence, and relatedness, they will generate higher-quality intrinsic motivation and sustained behavioral engagement (Deci and Ryan, 2000).

Therefore, under the joint perspective of the OST and the SDT, this study suggests that organizational support provides teachers with a good platform for growth, stimulates their sense of obligation to reciprocate, and simultaneously meets their three basic psychological needs of autonomy, competence, and relatedness, providing intrinsic psychological motivation for teachers to develop their autonomous learning ability and teaching ability. Teachers can continuously update their professional knowledge and adapt to the process of educational reform through their autonomous learning ability, while the improvement of teaching ability leads to observable teaching performance, such as students’ academic performance and teaching evaluation results. These professional growth and work performance effectively promote teachers’ recognition and professional approval from the organization. The above mechanisms jointly explain how organizational support ultimately leads teachers to gain professional recognition.

### 2.2 Organizational support and teacher professional recognition

Organizational support refers to the degree to which an organization values individual contributions and needs (Eisenberger et al., 1986). Recognition is a key motivational tool (van Woerkom et al., 2022) and a component in promoting professional identity (Pietarinen et al., 2013). Teacher professional recognition refers to the attainment of professional awards or encouragement, support, and appreciation from students, parents, colleagues, which is compatible with their work environment (Sullanmaa et al., 2024; Akinlar et al., 2025). A supportive school organization provides more platforms for teachers’ professional development, fostering a better environment for their professional growth (Yao et al., 2025). This encourages them to engage in their work with enthusiasm, dedication, and positive energy (Lipscomb et al., 2022), delve into professional development, and ultimately promote professional development and recognition. A recent empirical study of 2,956 primary and secondary school teachers in China found a significant positive correlation between teacher professional identity and perceived organizational support (*r* = 0.50, *p* < 0.01), providing large-sample empirical support for this study (Yu et al., 2025), which is conducive to further exploring the influence of organizational support on teachers’ professional recognition.

Therefore, this study proposed the following hypothesis:

*H*1: Organizational support positively influences professional recognition.

### 2.3 The mediating role of teachers’ autonomous learning ability

Attaching importance to autonomous learning activities is a hallmark of a high-quality education system (Serikov, 1991). Teachers’ autonomous learning ability refers to teachers’ high sense of ownership over their own learning, meaning they independently determine what they learn from the learning opportunities provided to them in the workplace (Admiraal et al., 2016). To reshape teachers’ professional development, awakening their growth needs and cultivating their awareness of proactive learning are key steps (Jing and Haiyang, 2018). This means that teachers’ professional identity and competence development are closely linked to their autonomous learning abilities.

On the one hand, to foster autonomous learning, school organizations must provide necessary support and professional development opportunities. SDT effectively explains how organizational support influences teachers’ autonomous learning abilities (Deci and Ryan, 2000). According to this theory, human motivation is categorized as intrinsic and extrinsic. Intrinsic motivation is determined by interest in the activity itself, while extrinsic motivation depends on the individual’s perception of the relationship between behavior and expected outcomes (such as tangible rewards; Gagné and Deci, 2005). Organizational support, by satisfying teachers’ three fundamental psychological needs for autonomy, competence, and relatedness (Hualing et al., 2024), can stimulate their enthusiasm for proactive exploration and, in turn, promote the development of their autonomous learning abilities (Kenesbekova et al., 2019). Previous research based on two rounds of longitudinal data (T1: 678 people, T2: 536 people) found that the organizational-level work resources experienced by teachers (including transformational leadership and colleague support) significantly and positively predicted the satisfaction of their basic psychological needs and had a positive impact on teachers’ professional learning commitment through autonomous motivation (de Jansen Wal et al., 2020), further confirming that organizational support can promote the cultivation of teachers’ autonomous learning ability.

On the other hand, teachers’ autonomous learning abilities are both a key characteristic of their professional identity and contribute to their professional recognition. Autonomy in their work is important, even necessary, for teachers to fulfill their professional tasks (Meyer, 2020). Research showed that active learning can promote teachers’ integration into their environment and gain professional recognition (Sullanmaa et al., 2024). Teachers’ active participation in professional training not only helps them create more diverse job opportunities but also earns them professional recognition from peers and superiors (Frase, 1989). In other words, Teachers’ autonomous learning ability positively predicts their professional recognition. Therefore, this study proposed the following hypothesis:

*H*2a: Organizational support positively influences teachers’ autonomous learning ability.

*H*2b: Teachers’ autonomous learning ability positively influences professional recognition.

*H*2: Autonomous learning ability positively mediates the relationship between organizational support and professional recognition.

### 2.4 The mediating role of teachers’ teaching ability

Teaching ability is an important component of teachers’ professional competence and the cornerstone of the profession. A teacher’s teaching ability is a specific ability, based on cognitive ability, that is demonstrated in specific subject teaching activities to achieve teaching objectives and successfully carry out teaching activities (Shen and Wang, 2000).

On the one hand, current research on the impact of organizational support on teachers’ teaching ability is inconsistent. For example, Smith et al. indicated that school support for junior high school mathematics teachers does not significantly improve teaching quality (Smith et al., 2018). However, Osmond-Johnson et al. pointed out that organizational support plays a vital role in enabling teachers to obtain high-quality professional learning experiences, thereby helping to improve their teaching ability (Osmond-Johnson et al., 2019). Based on the SDT (Deci and Ryan, 2008), this study suggests that when teachers receive teaching support from the organization, their needs for competence and relatedness are met, which gives them a sense of competence in teaching tasks and a sense of belonging as members of the teaching community, thereby motivating them to actively improve their teaching ability.

On the other hand, teachers’ teaching ability can promote their professional recognition. The three components of teachers’ teaching ability: teaching ability content knowledge, teaching enthusiasm, and self-regulation ability, all have a positive impact on teaching quality and student outcomes (Kunter et al., 2013), and these are key components for evaluating teachers’ teaching value. Professional recognition is closely related to these professional spirits (including continuous professional development and recognition of teaching value; Marques et al., 2020). In other words, when teachers’ teaching ability reaches a certain level and their teaching value is demonstrated, it will naturally help them gain professional recognition (Zhou, 2022). Based on the above research, this study proposed the following hypotheses.

*H*3a: Organizational support positively influences teachers’ teaching ability.

*H*3b: Teachers’ teaching ability positively influences professional recognition.

*H*3: Teachers’ teaching ability positively mediates the relationship between organizational support and professional recognition.

**Figure 1:**
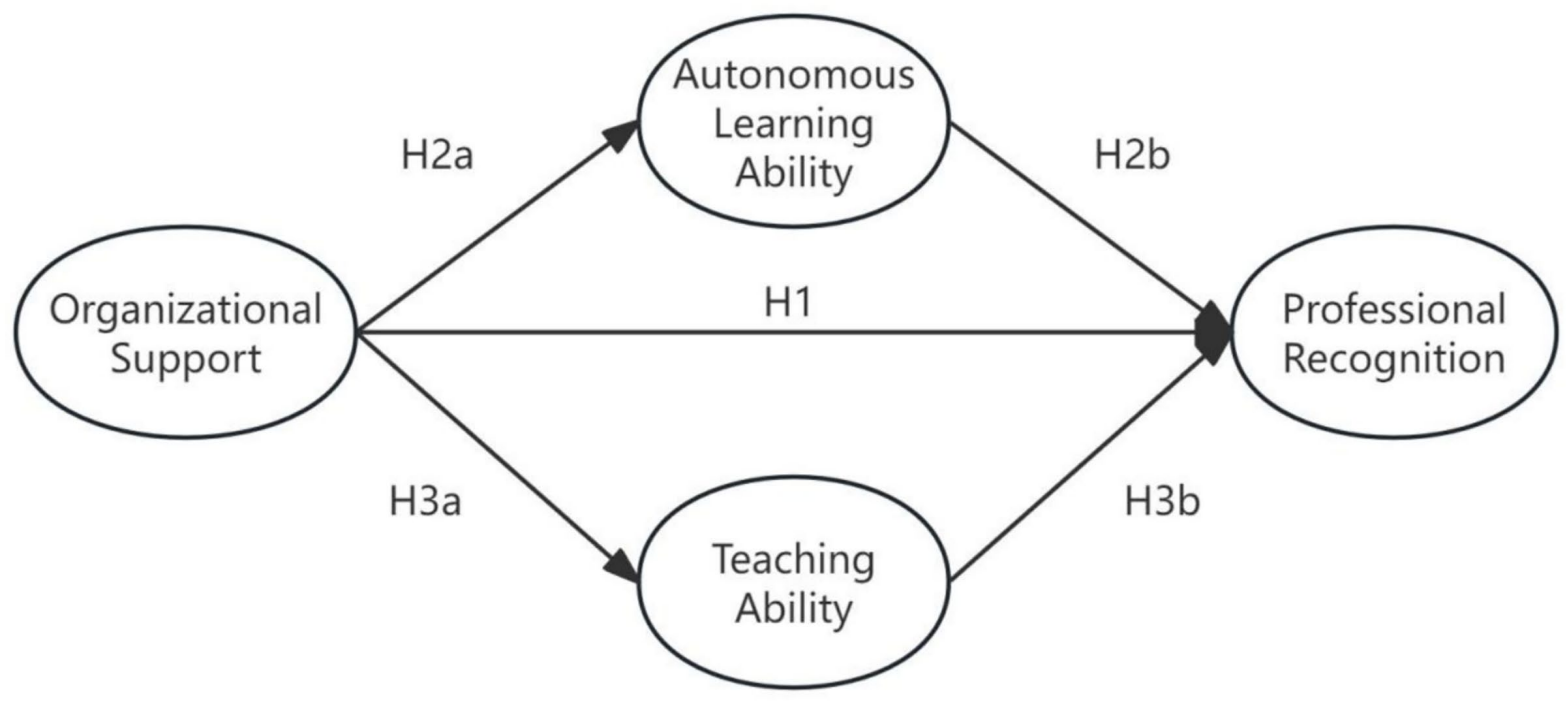
Conceptual framework.

In summary, this study constructed a parallel mediation model to explain the mechanism by which organizational support influences teachers’ professional recognition, as shown in Figure 1.

## 3 Materials and methods

### 3.1 Procedure and participants

This study employed convenience sampling to recruit teacher volunteers from 50 primary and secondary schools across 10 provincial-level administrative regions in China, including Beijing, Jiangsu, Guizhou, and Sichuan. Recruitment was conducted with the approval of school administrative departments. During the survey process, the research team obtained a complete list of all teaching staff from participating schools (a total of 5,761 teachers), then used a lottery system to select a certain number of teachers from each school as invitees, resulting in a total of 4,609 selected teachers. Selected teachers were informed that participation was entirely voluntary, and they had the right to withdraw at any time without facing any penalties. School administrators distributed electronic versions of the questionnaire to consenting teachers, who were required to complete it independently and truthfully without discussion, and then confirm and submit their responses. To protect respondents’ privacy, the questionnaire did not collect any personally identifiable information (such as names, phone numbers, or ID numbers).

Ultimately, based on the voluntary participation of the respondents, a total of 4,289 data entries were collected. After excluding invalid questionnaires, 3,983 valid responses were retained, yielding an effective response rate of 92.87%; among these respondents, 3,419 were female and 564 were male.

Furthermore, the study collected 3,983 valid responses, constituting a relatively large sample size. Utilizing a large sample characterized by regional diversity helps to enhance both the breadth and robustness of the research findings (Table 1).

**Table 1:** Demographic information (*N* = 3,983).

Items	N	%
Gender		
Male	3,419	85.8%
Female	564	14.2%
Degree		
Associate college or below	386	9.7%
Bachelor’s degree	3,439	86.3%
Master’s degree or above	158	4.0%
Age		
Under 20 years old	3	0.1%
20–25 years old	588	14.8%
26–30 years old	1,062	26.7%
31–40 years old	1,239	31.1%
41–50 years old	743	18.7%
51–55 years old	280	7.0%
Over 55 years old	68	1.7%
Years of Service as Teacher		
Under 6 years	1,585	39.8%
6–10 years	780	19.6%
11–15 years	470	11.8%
16–20 years	259	6.5%
21–25 years	357	9.0%
Over 25 years	532	13.4%
Provinces		
Eastern provinces such as Jiangsu and Shandong	1,682	42.2%
Central provinces such as Anhui and Hubei	1,336	33.6%
Western provinces such as Yunnan and Guizhou	965	24.2%

### 3.2 Research instruments

The research instrument consists of two parts. The first part contains sample demographic information, and the second part contains four scales: organizational support, autonomous learning ability, teaching ability, and professional recognition. These scales aim to explore factors influencing teachers’ professional recognition. The scales use a 5-point Likert scale, ranging from 1 (“strongly disagree”) to 5 (“strongly agree”). Completion is voluntary and anonymous to ensure the data accurately reflects teachers’ true feelings.

#### 3.2.1 Organizational support

This scale adopted the Organizational Support Scale used in the study by Rentao et al. (2013), which was adapted from the scale developed by Eisenberger et al. (2020) to fit the Chinese context. This scale has been applied in the Chinese context and has demonstrated good internal consistency (*α* = 0.874). It contains six items. For example, “When I encounter problems in my teaching work, I can seek help from the school.” The Cronbach’s alpha for this scale is 0.97.

#### 3.2.2 Teacher autonomous learning ability

This scale adapted the Teacher Autonomous Learning Ability Scale from the study by Acar et al. (2016). It consists of 11 items. An example item is “I can proactively set my own learning goals.” Its semantics had been adapted to better fit the Chinese context during translation. This scale is specifically designed for both in-service and pre-service teachers, and it closely aligns with the goal of this study, which focuses on teacher professional development. The Cronbach’s alpha for this scale is 0.983. With a Cronbach’s alpha coefficient greater than 0.7, it demonstrates strong reliability.

#### 3.2.3 Teacher teaching ability

This scale adapted the Teacher Teaching Ability Scale from the study by Di and Li (2023). It consists of 5 items. An example item is “I can innovate teaching methods and strategies to improve teaching effectiveness.” This scale captures teachers’ self-assessment abilities in key teaching areas and aligns with the focus of this study on teacher professional development. The Cronbach’s alpha for this scale is 0.941. With a Cronbach’s alpha coefficient greater than 0.7, it demonstrates strong reliability.

#### 3.2.4 Teacher professional recognition

This scale adopted the Teacher Professional Recognition Scale from the study by Sullanmaa et al. (2024). It consists of three items, such as “My colleagues encourage and support me.” Its semantics had been adapted to better fit the Chinese context during translation. This scale measures the teacher-centered teacher-work environment fit (Pietarinen et al., 2013), which can measure the professional recognition of the teacher group. The Cronbach’s alpha for this scale is 0.953. With a Cronbach’s alpha coefficient greater than 0.7, it demonstrates strong reliability.

### 3.3 Data analysis

A total of three software packages were utilized in this study: Mplus 8.0, SPSS 27.0 and Smart PLS 4.0. First, Mplus 8.0 software was used to test for common method bias. Second, SPSS 27.0 was employed to conduct descriptive statistics and correlation analysis. Third, Smart PLS 4.0 was used to evaluate the measurement model. Finally, Smart PLS 4.0 was used to construct a structural equation model incorporating parallel mediation effects, with “organizational support” as the independent variable, “teacher professional recognition” as the dependent variable, and “teacher autonomous learning ability” and “teacher teaching ability” as parallel mediation variables. The model was then evaluated. Smart PLS 4.0 employs Partial Least Squares Structural Equation Modeling (PLS-SEM) to analyze data. PLS-SEM was chosen because of its high adaptability and strong statistical power, which enables it to demonstrate robust and efficient performance in large sample estimation, and it can handle complex variables and structural paths (Hair et al., 2019).

## 4 Results

### 4.1 Common method bias

This study used Mplus 8.0 to conduct a Confirmatory Factor Analysis (CFA) to test for common method bias. CFI ≥ 0.95, TLI ≥ 0.95, RMSEA ≤ 0.06, SRMR ≤ 0.08 indicate relatively good fit (Hu and Bentler, 1999). The results indicated that the one-factor model had a poor fit (RMSEA = 0.207, CFI = 0.686, TLI = 0.657, SRMR = 0.101), thus suggesting that there was no serious common method bias (Iverson and Maguire, 2000; Podsakoff et al., 2003).

### 4.2 Correlation analysis

This study used SPSS 27.0 to conduct descriptive statistics and correlation analysis. Table 2 lists the bivariate correlations for the four variables. The mean score for each variable represents the participants’ level of agreement with the survey questions. The correlation coefficients were interpreted using traditional effect size criteria, where values of approximately 0.10, 0.30, and 0.50 are typically considered small, medium, and large correlations, respectively (Cohen, 1988). Overall, all variables showed significant positive correlations. Therefore, the observed positive correlations provide preliminary support for the hypothetical relationship between organizational support, autonomous learning ability, teaching ability, and professional recognition.

**Table 2:** Descriptive statistics and correlations.

Variable	Mean	SD	1	2	3	4
1 PR	4.093	0.787	1			
2 TA	3.827	0.858	0.689**	1		
3 ALA	4.258	0.685	0.760**	0.681**	1	
4 OS	4.024	0.884	0.758**	0.603**	0.698**	1

**p < 0.01; PR, Professional recognition; TA, Teaching ability; ALA, Autonomous learning ability; OS, Organizational support.

### 4.3 Measurement model evaluation

This study used Smart PLS 4.0 to evaluate the measurement model. As shown in Table 3, the Cronbach’s alpha coefficient and Composite reliability values for all constructs exceeded the recommended threshold of 0.70, indicating good internal consistency reliability. Furthermore, the mean variance extracted (AVE) values were all above 0.50, indicating good convergent validity (Hair et al., 2019). Discriminant validity was supported because the square root of the AVE for each construct was greater than its correlation coefficient with other constructs. In addition, the HTMT values were below the recommended thresholds of 0.85, further indicating acceptable discriminant validity (Henseler et al., 2015).

**Table 3:** Reliability, convergent validity, and discriminant validity of the model.

Variable	Cronbach’s alpha	Composite reliability (rho_a)	Composite reliability (rho_c)	AVE	Discriminant validity (HTMT)			
					1	2	3	4
1 PR	0.953	0.953	0.969	0.914	-			
2 TA	0.941	0.947	0.955	0.809	0.739	-		
3 ALA	0.983	0.983	0.985	0.854	0.786	0.726	-	
4 OS	0.97	0.97	0.976	0.87	0.791	0.642	0.718	-

PR, Professional recognition; TA, Teaching ability; ALA, Autonomous learning ability; OS, Organizational support.

### 4.4 Structural model evaluation

**Figure 2:**
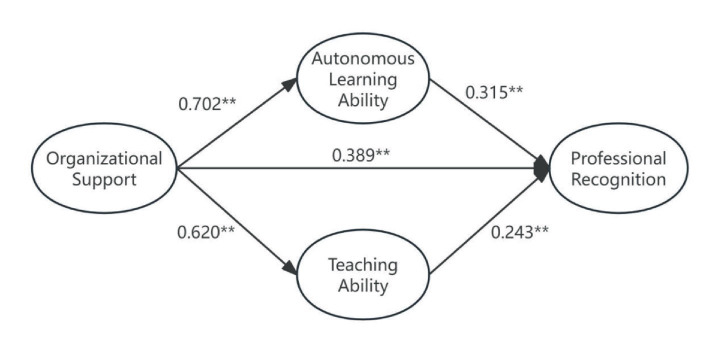
Parallel mediator model. **p < 0.01.

This study used Smart PLS 4.0 to test the fit of the structural equation model. A structural equation model was constructed with teachers’ organizational support as the predictor variable, professional recognition as the outcome variable, and autonomous learning ability and teaching ability as mediating variables. As a guideline, the R^2^ values of 0.75, 0.50, and 0.25 can be considered substantial, moderate, and weak (Hair et al., 2011). The coefficients of determination (R^2^) for ALA, TA, and PR were 0.493, 0.385, and 0.707, respectively. The total explanatory power of the model was 0.707, indicating that the model had strong predictive ability for PR. The structural equation model is shown in Figure 2.

### 4.5 Hypotheses testing results

Figure 2 and Table 4 present the results of the hypothesis tests. Organizational support (*β* = 0.389, *p* < 0.001) has a significant positive impact on teachers’ professional recognition, verifying H1. Additionally, organizational support has significant and positive effects on teachers’ autonomous learning ability (*β* = 0.702, *p* < 0.001) and teaching ability (*β* = 0.620, *p* < 0.001), verifying H2a and H3a. This study constructed two mediating variables: teachers’ autonomous learning ability and teaching ability. Both teachers’ autonomous learning ability (*β* = 0.315, *p* < 0.001) and teaching ability (*β* = 0.243, *p* < 0.001) have significant positive impacts on teachers’ professional recognition, verifying H2b and H3b.

**Table 4:** Hypotheses testing.

Path	Path coefficient	STDEV	95% confidence intervals	T statistics	*p* values	Significance
Direct effects						
H1 OS → PR	0.389	0.023	[0.344,0.434]	17.195	0.000	Yes
H2a OS → ALA	0.702	0.012	[0.678,0.725]	57.964	0.000	Yes
H3a OS → TA	0.620	0.013	[0.594,0.646]	47.072	0.000	Yes
H2b ALA→ PR	0.315	0.022	[0.272,0.359]	14.282	0.000	Yes
H3b TA → PR	0.243	0.018	[0.207,0.277]	13.835	0.000	Yes
Indirect effects						
H2 OS→ALA→TPR	0.221	0.017	[0.189,0.254]	13.225	0.000	Yes
H3 OS → TA → TPR	0.150	0.011	[0.128,0.173]	13.439	0.000	Yes
Total indirect effects						
OS → PR	0.371	0.017	[0.337,0.404]	21.235	0.000	Yes

## 5 Discussion

### 5.1 The direct effect of organizational support on professional recognition

In this study, organizational support can directly predict teachers’ professional recognition, which further validates previous research (Wayne et al., 2002). This result indicates that teachers’ professional recognition is closely rooted in the organizational environment of the school. Specifically, when schools provide professional development opportunities, administrative support, emotional care, and collaboration among colleagues to teachers, they are more likely to feel that their work is valued and recognized (Eisenberger et al., 2020). Meanwhile, based on the Status Characteristics Theory (Berger et al., 1972), the allocation of organizational support resources forms colleagues’ interpretation tendencies of teachers’ professional capabilities. By granting teachers leadership roles within the organization (Allen, 2018) and providing policy support (Sarmurzin, 2024), their status can be continuously enhanced, thereby strengthening their authority, initiative, and recognition among colleagues (Carl et al., 2022). That is, the more the organization supports a teacher’s development and elevates them to a core position in the work, the more it directly affects colleagues’ recognition and appreciation of their professionalism.

However, it is worth noting that teachers who receive more professional recognition may have more opportunities to obtain resource allocation and leadership attention from the school, hold a positive view of the school environment, and thus report higher levels of organizational support (Kiiza et al., 2025), indicating a possible reinforcing cycle of “support-recognition-support” that merits further investigation. Meanwhile, although this effect is highly significant statistically, from the perspective of effect size, it is only at a medium level (Cohen, 1988), indicating that organizational support is not the only or decisive factor for professional recognition. Further examination of factors such as individual ability is needed to understand their impact on obtaining professional recognition.

### 5.2 The mediating role of teachers’ autonomous learning ability

In this study, teachers’ autonomous learning ability played a key mediating role between organizational support and professional recognition, providing an important analytical perspective for understanding how teachers internalize organizational support.

Firstly, organizational support can significantly promote teachers’ autonomous learning ability, which further validates previous research (Power and Goodnough, 2019). The path effect size is strong, indicating that a supportive school environment may be particularly effective in stimulating teachers’ self-directed professional development. From the perspective of SDT, organizational support may meet teachers’ needs for autonomy, competence, and relatedness, thereby encouraging them to set learning goals, seek professional resources, and engage in reflective practice (Deci and Ryan, 2008). Recent empirical research on K-12 teachers in China has further demonstrated that autonomous support, competence support, and relational support significantly predict intrinsic motivation, with competence support having the strongest effect. Intrinsic motivation, in turn, strongly predicts continuous learning behavior (Chen et al., 2026). These findings are of theoretical significance because it suggests that the role of organizational support is not limited to being an external condition; it may also be transformed into teachers’ internal development motivation, thereby enhancing their learning ability within the organization (Seashore Louis and Lee, 2016).

Secondly, autonomous learning ability can significantly promote professional recognition, which differs from the study of Xu et al. (2024). The latter focused on the online learning situation of college students during the pandemic and suggested that fostering students’ recognition of their future professional identities could enhance their autonomous learning ability. That is, there may be a dynamic reciprocal mechanism between autonomous learning ability and professional recognition. However, due to the cross-sectional design of this study, this dynamic relationship cannot be clearly determined. Future longitudinal studies are needed to examine whether teachers’ autonomous learning ability increases professional recognition over time or whether existing professional recognition also affects their autonomous learning ability.

Furthermore, there may be significant group differences in teachers’ autonomous learning ability. Empirical research from China indicates that gender, age, and the grade level taught all have systematic effects on teachers’ autonomous learning levels (Wang et al., 2022). This finding suggests that in advocating for teachers’ autonomous learning and professional development, education system should abandon a “one-size-fits-all” approach and instead adopt more precise and differentiated support strategies to maximize the learning potential of all teachers.

### 5.3 The mediating role of teachers’ teaching ability

In this study, teachers’ teaching ability also played a key mediating role between organizational support and professional recognition.

On the one hand, organizational support can significantly enhance teachers’ teaching ability, which further validates previous research (Chen et al., 2025). Teachers who feel strongly supported by the organization tend to have higher self-efficacy (Jiao et al., 2022), higher job satisfaction (Crucke et al., 2022), and higher organizational commitment (Eisenberger et al., 2020), and these psychological resources all contribute to improving teaching performance (Li et al., 2025). A supportive environment promotes teachers’ professional growth, enabling them to try innovations in teaching and conduct more effective classroom interactions (Han et al., 2021), thereby continuously enhancing their teaching competence in practice.

On the other hand, teachers’ teaching ability can significantly foster their professional recognition, which also further validates previous research (Zhou, 2022). In most school environments, the professional value of teachers is often judged by visible teaching performance, classroom effectiveness, student development outcomes, and the extent of participation in teaching-related activities (Cohen and Goldhaber, 2016; Bacher-Hicks et al., 2019). Teachers with excellent teaching competence (such as subject-specific pedagogical knowledge, teaching enthusiasm, and self-regulation skills) can consistently demonstrate higher teaching quality, which not only promotes students’ academic performance and learning motivation (Kunter et al., 2013) but also enhances their professional reputation among colleagues, students, and administrative managers (Ivanchuk et al., 2020), thereby solidifying their professional image in the educational community. However, this mechanism also reflects a certain performance-oriented cultural atmosphere within schools, where the degree of recognition a teacher receives largely depends on observable teaching outcomes. While this performance-oriented culture can motivate teachers to improve their teaching performance in the short term, it may also lead to the neglect of invisible labor and long-term educational values in the teaching process (Staudt Willet and He, 2024). Therefore, while schools should affirm teaching achievements, they should also establish a diverse and developmental evaluation system to balance outcome-oriented and process-oriented evaluations.

By comparing the two mediating paths, the mediating effect of autonomous learning ability was 0.221, accounting for 60% of the total indirect effect, while the mediating effect of teaching ability was 0.150, accounting for 40% of the total indirect effect. This indicates that organizational support mainly enhances teachers’ professional recognition by empowering their self-directed learning rather than relying primarily on the improvement of explicit teaching skills. In other words, the organizational support perceived by teachers is more likely to activate their intrinsic learning motivation and autonomous development ability rather than merely improving observable teaching performance. This finding has important implications for school management practices: if managers overly focus on teaching skill training (such as open classes and teaching competitions), while relatively neglecting the systematic cultivation of teachers’ autonomous learning motivation and ability (such as providing learning resources, ensuring time for self-study, and building learning community support), it may not be conducive to teachers’ continuous growth and professional advancement in the long run. Particularly alarming is that as teaching experience accumulates, teachers lacking an internal drive for autonomous learning are prone to burnout (Brouhier et al., 2026), losing enthusiasm for teaching and professional development, and thus finding it difficult to maintain vitality and innovation (Huang et al., 2025).

### 5.4 Implications

Theoretically, this study was based on the organizational support theory and self-determination theory. Through large-scale empirical data, it revealed the dual mediating paths of organizational support influencing teachers’ professional recognition, making significant expansions to the existing theoretical framework.

First, it constructed an integrated analytical framework of “external resources → personal ability → professional recognition”. This study incorporated organizational support, autonomous learning ability, and teaching ability into the same model and found that organizational support not only has a significant positive direct effect on teachers’ professional recognition but also exerts its influence through two indirect paths. This result validated and enriched the applicability of the organizational support theory in the field of education, while also expanding the content of the self-determination theory regarding “the satisfaction of basic psychological needs promoting intrinsic motivation and behavioral output” (Deci and Ryan, 2000). Second, it identified and compared the differentiated contributions of the two ability mediating paths. The research found that the mediating effect of autonomous learning ability is slightly higher than that of teaching ability, and both paths are partial mediations. This indicates that the influence of organizational support on teachers’ professional recognition is achieved not only through empowering teachers’ awareness of autonomous development (autonomous learning ability) but also through enhancing their classroom teaching practice level (teaching ability). In the process of transforming organizational support into professional recognition, teachers’ active learning and self-directed initiative may have a stronger explanatory power than visible teaching performance. This finding makes up for the deficiency of previous studies that did not distinguish between different ability mediating mechanisms, providing a more detailed theoretical perspective for understanding the multiple paths of teachers’ professional recognition formation.

Practically, this study provided guidance for enhancing teacher professional recognition. First, schools should not merely offer formal and fragmented support but should establish a comprehensive teacher support system, including institutional support, leadership support, and peer support, to create a synergistic support force, continuously follow up, and enhance teachers’ sense of organizational belonging and well-being. Furthermore, in terms of professional development, schools can provide teachers with relevant training opportunities and platforms, such as encouraging them to participate in school teaching decision-making, inter-school exchange activities, and teaching innovation competitions, to promote the development of teacher identity and professional growth (Assor et al., 2020). Second, the education system should establish differentiated support mechanisms. Targeted support strategies should be designed for teachers at different development stages, subject backgrounds, and school types. For example, resource support should be strengthened for teachers in rural schools, while support for STEM teachers should focus on updating teaching techniques. Third, schools need to innovate their evaluation and incentive mechanisms. Teachers’ professional recognition can be incorporated into school evaluation systems, while also praising and rewarding teachers’ efforts in teaching innovation and professional development to stimulate their work enthusiasm.

## 6 Limitations and future research

This study also had certain limitations. First, the study primarily employed a cross-sectional design, making it difficult to confirm causal relationships between variables. Future studies should employ longitudinal follow-up studies or intervention experiments for further verification. Second, the sample coverage was limited. The research subjects are primary and secondary school teachers from 10 provinces. The diversity and representativeness of the samples still need to be further improved. Future studies should expand the geographical diversity of the sample to deepen the promotion work nationwide. Finally, this study mainly used the questionnaire survey method. Although the sample size is large, there may still be errors. Future studies can combine multiple methods such as behavioral observation and in-depth interviews for evaluation.

## 7 Conclusion

Teacher professional recognition is crucial for building a high-quality teaching team. This study explored the mechanism through which organizational support influences teachers’ professional recognition. The results indicate that organizational support can effectively enhance teachers’ professional recognition. Moreover, both autonomous learning ability and teaching ability play significant mediating roles in this relationship. These findings suggest that organizational support not only directly boosts teachers’ sense of professional recognition but also indirectly strengthens it by enhancing their self-directed learning ability and teaching ability. These discoveries highlight the importance of organizational support and personal capabilities in the process of teachers achieving professional recognition, which can improve educational outcomes and teachers’ well-being.

This study constructed and empirically verified the mechanism connecting organizational resources, teachers’ professional abilities, and professional recognition, enriching the existing theoretical knowledge system and expanding the application scope of organizational support theory and self-determination theory. Additionally, the study proposed suggestions. Schools should establish a regular support system, such as setting up a teacher development fund, improving the mentorship system, and conducting regular caring communication, to make teachers truly feel the support from the system, leaders, and colleagues. Secondly, differentiated support strategies should be implemented for different types of schools and subjects, and support strategies should be flexibly adjusted. Finally, the teacher evaluation and incentive mechanism should be innovated, timely recognizing teachers’ progress and achievements, and truly enhancing the concern and importance given to teachers, thereby promoting the construction of an educational power.

Future research could adopt longitudinal or experimental designs, expand sample diversity and representativeness, and integrate qualitative evidence to enhance the validity and robustness of research results.

## Data Availability

The raw data supporting the conclusions of this article will be made available by the authors, without undue reservation.
